# Autonomous sampling and SHAP interpretation of deposition-rates in bipolar HiPIMS

**DOI:** 10.1039/d6dd00063k

**Published:** 2026-04-27

**Authors:** Alexander Wieczorek, Nathan Rodkey, Jan Sommerhäuser, Jason Hattrick-Simpers, Sebastian Siol

**Affiliations:** a Laboratory for Surface Science and Coating Technologies, Empa–Swiss Federal Laboratories for Materials Science and Technology Ueberlandstrasse 129 Duebendorf CH-8600 Switzerland nathan.rodkey@empa.ch sebastian.siol@empa.ch; b Department of Materials Science and Engineering, University of Toronto M5S 3E4 Toronto ON Canada; c Acceleration Consortium, University of Toronto M7A 2S4 Toronto ON Canada

## Abstract

High-power impulse magnetron sputtering (HiPIMS) offers considerable control over ion energy and flux, making it invaluable for tailoring the microstructure and properties of advanced functional coatings. However, compared to conventional sputtering techniques, HiPIMS suffers from reduced deposition rates. Many groups have begun to evaluate complex pulsing schemes to improve upon this, leveraging multi-pulse schemes (*e.g.* pre-ionization or bipolar pulses). Unfortunately, the increased complexity of these pulsing schemes has led to high-dimensionality parameter spaces that are prohibitive to classic design of experiments. In this work we evaluate bipolar HiPIMS pulses for improving deposition rates from Al and Ti sputter targets. Over 3000 process conditions were collected *via* autonomous Bayesian sampling over a 6-dimensional parameter space. The resulting machine-learning model was then interpreted using Shapley Additive Explanations (SHAP), to deconvolute complex process influences on deposition rates. This allows us to link observed variations in deposition rate to physical mechanisms such as back-attraction and plasma ignition. Insights gained from this approach were then used to target specific processes where the positive pulse components were expected to have the highest impact on deposition rates. However, in practice, only minimal improvements in deposition rate were achieved. In most cases, the positive pulse appears to be detrimental when placed immediately after the neg. pulse which we hypothesize relates to quenching of the afterglow plasma. The proposed workflow combining autonomous experimentation and interpretable machine learning is broadly applicable to the optimization of complex plasma processes, paving the way for physics-informed, data-driven advancements in coating technologies.

## Abstract 2

Autonomous loops and SHAP analysis allow for comprehensive, statistical analysis of >3000 different bipolar HiPIMS processes.

## Graphical abstract

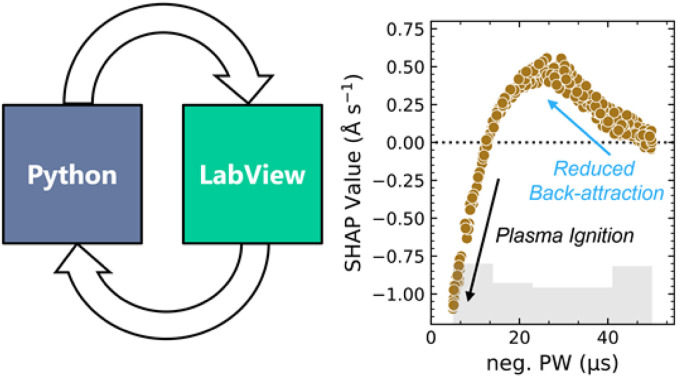

## Introduction

High-power impulse magnetron sputtering (HiPIMS) is a deposition technique where short, µs-scale voltage pulses are applied to a sputter target at low duty cycles. Consequently, high peak-current densities (*J*_pk_) and higher plasma-densities can be achieved, resulting in a higher ionized flux fraction of the sputtered species.^1^ This leads to some unique advantages, as increased ionization allows for an accelerating substrate bias to be applied, which can be used to tailor microstructure, stress, and even phase formation.^2–7^ Moreover, the generally higher kinetic energy of species can be leveraged to improve adatom mobility, leading to dense, highly oriented structures, as shown recently for AlN and AlScN thin films.^8–10^ However, HiPIMS processes suffer from low deposition-rates when compared to their DC or pulsed-DC analogues.

Several factors contribute to this low deposition rate, one of the most important being ion back-attraction; a phenomenon by which positively charged ions are attracted to the negatively charged sputter target, reducing their probabilities for escape.^11^ Additionally, the low duty cycles common in HiPIMS processes mean that significant energy goes into ionizing the process gas at the beginning of each pulse.^1,5,7^ As a result, improving deposition-rates has been of particular interest to the community, studied through a variety of techniques and methods. Popular among these is the introduction of different pulsing schemes, such as the use of mid-frequency pulses to pre-ionize the plasma,^12,13^ pulse-packeting,^14–16^ or bipolar signals.^17^ This growing complexity mirrors a broader trend in materials processing. Unfortunately, the amount of data available in the field for these techniques is still limited and increasing pulse complexity makes classical design of experiments prohibitive.

Specifically, in the case of bipolar HiPIMS pulses, recent reports have shown a notable increase in the deposition-rate of sputtered ions. This was attributed to the idea of a positive sheath around the sputter target that would reflect ions and reduce back-attraction.^18,19^ However, these results were contradicted by studies that failed to see any experimental increase in deposition rate,^20–22^ as well as several studies of plasma dynamics that showed that the initial idea of an ion-reflecting sheath was not seen experimentally.^17,20,21,23^ This was related to similar contradictory reporting in bipolar HiPIMS of ion acceleration onto insulating substrates which relied on the idea of this ion-reflecting sheath. Previously, Tiron *et al.*^24^ demonstrated that ion acceleration towards a floating substrate is possible using bipolar pulses with proper balancing of the magnetrons; in most other cases the positive component of the pulse increases the bulk plasma potential, dropping only as it approaches the substrate sheath.^17,20–23,25,26^ In such cases, no notable ion acceleration is observed when the surface potential closely matches that of the plasma.

Such contradictory reporting can partly be explained by the complexity of the process when configuring pulse shapes. A bipolar HiPIMS pulse adds three parameters to the dimensionality of the process (*i.e.* pos. delay, pos. pulse-width, and pos. voltage). Coupled with typical process adjustments to frequency, pulse-width, and peak-current density (*J*_pk_) of the negative pulse, the number of permutations in the experimental design make process development exceedingly difficult through a classic design of experiments. In response to this, most reports on bipolar HiPIMS fix two of these three features, changing only the voltage or delay in their experimental design.^18,19,26^ As such, to unlock the full potential of bipolar HiPIMS, there is a need for larger datasets, coupled with an efficient way to collect and interpret these datasets.

Data rich experimentation and machine learning (ML) approaches promise to solve this timely challenge, aiming to accelerate understanding and control of processes with increasing complexity. In many fields, rapid adoption of experimental automation and ML techniques has already materialized this potential.^27–35^ However, the development of self-driving or autonomous physical vapor deposition (PVD) systems has generally lagged behind, with few examples of fully autonomous PVD labs.^32^ This can be attributed to the complexity involved in experimental automation of vacuum systems as well as the need for *in vacuo* materials characterization. A more practical approach to accelerated PVD process development is to use *in situ* process diagnostic which allows for non-intrusive, continuous data collection^36–38^ and/or batch processing.^39,40^ Generally, these experimental setups rely on Bayesian optimization to efficiently sample process windows, however, high-dimensionality processes (*e.g.* bipolar HiPIMS) require additional, complex interpretation tools.

Fortunately, developments in ML interpretation published in 2017 by Lundberg *et al.* showed that Shapley Additive Explanations (SHAP), a statistical formulation originating from game theory, could be used to interpret feature influence in complex machine-learning models.^41^ SHAP explanations work by calculating the expected contribution of each feature (*e.g.* process parameter) to an output (*e.g.* deposition rate), shown as a deviation from the model's base value (*i.e.* the mean prediction). This is also referred to as the marginal contribution of the given feature and has the same physical units as the model output, enabling quantitative interpretation of feature effects. SHAP has become a widely adopted method for model interpretability and has been increasingly applied to process design in materials science.^42–49^

In this work, we use Bayesian optimization to efficiently sample the deposition-rate in a bipolar HiPIMS discharge and use SHAP to interpret the resulting datasets. The resulting dataset represents the deposition-rates of >3000 process conditions for bipolar HiPIMS pulses. We choose Al and Ti metals for the study to compare materials with relatively lower (*e.g.* Al) or higher (*e.g.* Ti) ionized flux fraction. In addition, Ti typically exhibits higher amounts of doubly, or triply charged ions (*e.g.* Ti), which we expect to have stronger interactions with the positive pulse components.^50^ SHAP-interpretation of the data demonstrated that generally, the positive components of a bipolar HiPIMS pulse have no meaningful impact on the deposition-rate, as proven for both Al and Ti datasets. Subsequently, we targeted specific processes in a high *J*_pk_ dataset where the positive pulse components were expected to have the largest impact, however, potential gains in deposition rate were minimal. Instead, many processes showed reduced deposition rates with the introduction of a pos. pulse, which we hypothesize relates to quenching of the afterglow plasma. Finally, the large amount of data represented in this work allow for a powerful visual of back-attraction and plasma ignition effects in HiPIMS as they relate to deposition rates.

## Experimental methods

**Fig. 1:**
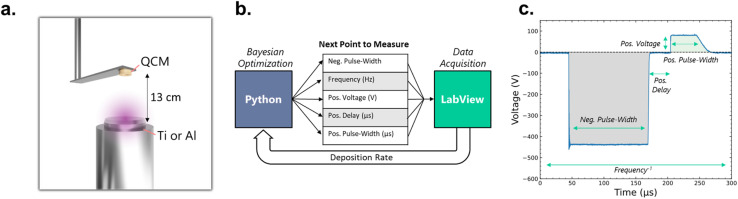
Schematic of (a) the experimental setup, involving a QCM placed at a distance of 13 cm from a Ti or Al sputter target, (b) the feedback loop used between a Python-based Bayesian optimization algorithm, which communicates with LabView to run different process parameters, and (c) an example of a bipolar HiPIMS pulse.

Experiments were carried out in a custom-built sputter chamber using an unbalanced 3″ magnetron (A330, AJA International Inc.) in a coplanar, sputter-up geometry. The sputter targets were sourced from Kurt J. Lesker at a purity >99.99% for both Al and Ti targets. All depositions were performed with 50 sccm Ar flow, regulated to 0.5 Pa working pressure by throttling a gate-valve. A quartz-crystal monitor (QCM) was used to measure deposition rates, placed at a distance of 13 cm from the sputter target (shown schematically in Fig. 1a). Datasets were collected using constant power on the sputter target, requiring multiple datasets to be collected at different power densities (2.63, 4.4, and 5.48 W cm^−2^) to access high peak-current density (*J*_pk_) over a wide pulse-width range of 5–300 µs. The boundary conditions and accessible *J*_pk_ range for each of these datasets is summarized in Table S1. In short, a low and high negative pulse-width (neg. PW) was collected for both Al and Ti, constrained to frequencies between 500–5000 Hz for the low neg. PW datasets (5–100 µs), and frequencies of 200–800 Hz for high neg. PW datasets (100–300 µs). Additionally, a low duty-cycle dataset was collected for both Al and Ti to access higher *J*_pk_ (1.5–1.8 Acm^−2^), constrained to a PW of 5–50 µs and a duty cycle (frequency × PW) range of 1.2–3.75%. Additionally, to prevent overlapping between the positive and negative pulse components, the lower and upper bounds of the pos. delay and PW components were chosen accordingly, set to a range from 0–40 µs.

The sputter chamber, workflow, and an example of a bipolar HiPIMS pulse pattern are shown schematically in Fig. 1. The sputter chamber is controlled *via* software implemented in LabView 2024 Q1, which interfaces with Python 3.12.11 for the adaptive sampling. This sampling uses Bayesian optimization implemented through the open-source package BayBE 0.13.1 which is based on BoTorch 0.14.0.^51^ A Gaussian Process Regressor (GPR) based on a Matérn kernel was used as the surrogate model. As default in BayBE, hyperparameters were optimized using the L-BFGS-B algorithm *via* the fit_gpytorch_mll routine from BoTorch.^52^ The GPR models for each campaign were also k-fold cross-validated, showing moderate accuracy with an *R*^2^ of 0.61–0.82. The cross-validation results can be found on the Github repository. The Bayesian optimization algorithm was run in exploration mode by using the posterior standard deviation to minimize uncertainty within the predefined bounds of the parameter-space. Once the process conditions are set by the Python routine, LabView executes it, first stabilizing the process for 10 s, followed by measurement of the deposition-rate, done by integrating the total deposition until 35.1 and 21.7 Å were deposited for Al and Ti respectively (total mass deposited on the QCM was the same for both Al and Ti). The corresponding process parameters are then logged, including the oscilloscope waveforms of each process, averaged over 10 scans. Peak-currents are then determined from the waveforms using a *Python* algorithm. All *Python* code used in this work is included in the SI.

The deposition rate of >3000 processes were then interpreted using Shapley Additive Explanations (SHAP).^41,53^ All SHAP interpretations shown in this work are done using an Exact Explainer unless otherwise stated; interfaced *via* an open-source package, SHAP version 0.50.0.^53^ The GPR model used in the exploration was kept for SHAP analysis; a comparison of the GPR model to a random forest and linear regression are included in the GitHub repository. The GPR performed similar to a random forest and significantly better than linear regression. Interpretations of the dataset presented in this work were also done with linear model agnostic explanations (LIME), which identified the same dominant features in the datasets, but failed to explain some of the complex, nonlinear trends found in this work. The full comparison to LIME is included in the GitHub repository, as well as both scatter plots and feature importance of an example Al, short PW dataset (Fig. S1 and S2). Notably, *J*_pk_ was not controlled in the Bayesian optimization (only measured), and the uncertainty was thus not minimized across that space. The uncertainty across the parameter space was evaluated, including *J*_pk_, by calculating the mean standard deviation over a uniformly spaced mesh of ∼390 000 points, shown in Fig. S3 for all 6 datasets. The mean standard deviation does not reduce significantly past 100 measurements.

## Results and discussion

### Global overview of data

**Fig. 2:**
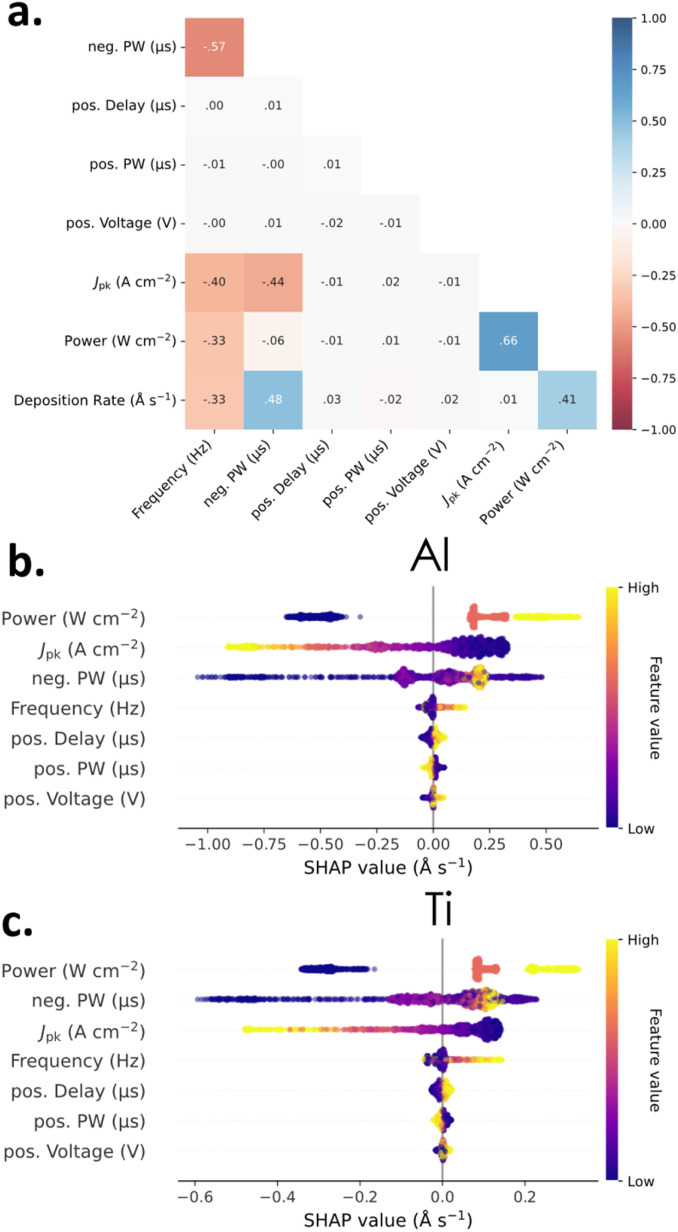
Global overview of the bipolar HiPIMS data sets. (a) Spearman correlation matrix of all process parameters as well as the resulting deposition rate. For (b) Al, and (c) Ti, a SHAP beeswarm plot with the mean of the datasets marked as a solid line, and the SHAP value on the *x*-axis indicating the predicted deviation from the model mean caused by each process parameter. Process parameters are ordered by feature importance.

We start our investigation with a global analysis of all datasets recorded in this study. Notably, this includes studies on both Al and Ti metal targets. Analysis of the Spearman correlation coefficients, as shown in Fig. 2a, suggests low pairwise correlation between most process parameters. Additionally, individual spearman correlation matrices for Al and Ti only datasets are shown in Fig. S4. Of note, power density showed a high correlation value of 0.73 with *J*_pk_ but was left in the analysis to allow for a generalized overview of the six datasets, which were performed at different power densities. Following this, SHAP was used to estimate feature importance.

This global overview is shown in Fig. 2b and c as beeswarm plots, summarizing the three datasets of Al in Fig. 2b and of Ti in Fig. 2c. In a beeswarm plot, features are ranked by importance according to the absolute sum of their SHAP values, with the model mean set to zero, shown as a black reference line. Each point represents a unique process, for which the SHAP values are the attributed deviation from the model mean caused by that parameter. The sum of all SHAP values in a process sum to 0 and SHAP values retain the units of the model output (Å s^−1^).

Among all parameters, power density emerges as the most influential factor driving deposition rate. However, the broad spread of SHAP values indicate overlapping effects and limited separability between their contributions; an expected outcome given the sparse sampling of power density values. Following the power density, unipolar HiPIMS process parameters (*J*_pk_, neg. PW, and frequency) rank next in feature importance. Trends observed in the unipolar components are consistent with established observations. High values of *J*_pk_, drop the deposition rate, attributed to the correlation of high *J*_pk_ to higher ion counts and a corresponding reduction in deposition rates from the increased influence of back-attraction of these ions. Next, the neg. PW trends are more nuanced, suggesting some local maximum, with the highest contributions to deposition-rate coming from lower neg. PW. This is also an expected reaction relationship relating to back-attraction as low neg. PW values help mitigate back-attraction effects, as shown by Shimizu *et al.*^11^ This relationship is discussed in greater detail in the following section. The frequency has the smallest effect amongst the unipolar pulse parameters, showing an expected increase in deposition rate at higher frequency values. This is also a well-reported phenomenon, where the higher background plasma densities at higher frequencies allow for more efficient plasma ignition and greater sputter yield.^12,13,54^ Finally, parameters associated with the pos. pulse components of bipolar HiPIMS display low absolute SHAP values, suggesting only a minor contribution to the overall deposition rate. Individual beeswarm plots for all six datasets are provided in Fig. S5.

The deposition landscape is also visualized by 2D cross sections in Fig. S6 and S7. These plots show how the model predicts the deposition rate across a 2D slice of frequency and neg. PW (Fig. S6) and *J*_pk_ and neg. PW (Fig. S7). Overall, the trends mirror the conclusions in the global beeswarm and reinforce trends found in the SHAP analysis presented later in this work.

### Interactions and correlations

**Fig. 3:**
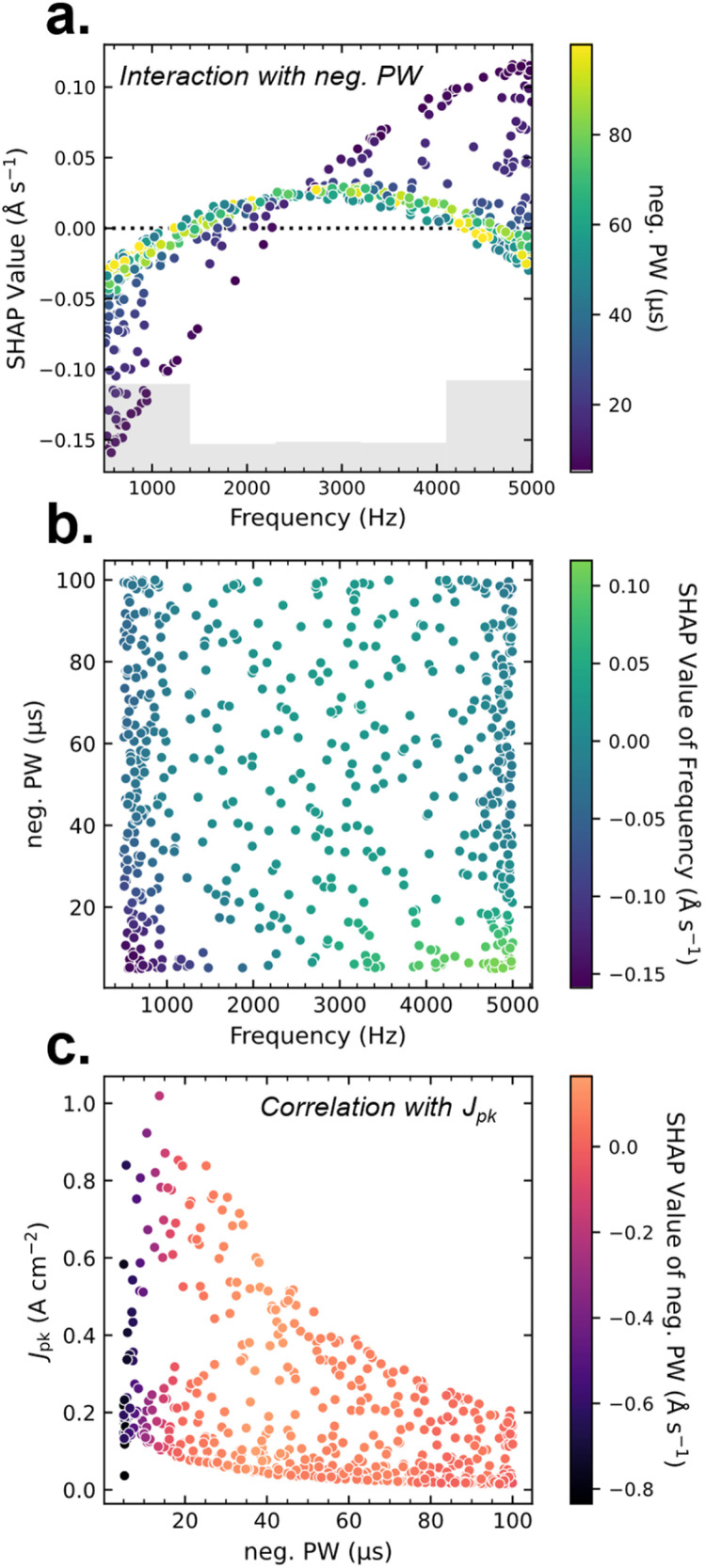
SHAP analysis for a low pulse-width, Al dataset is shown above. In (a) dependence plot, highlighting an interaction effect between frequency and neg. PW. The model mean of the dataset is marked as a dotted line. The distribution of data collected over the parameter space is visualized by a grey bar chart. The distribution of sampled points is then shown for (b) neg. PW *vs.* frequency and (c) *J*_pk_*vs.* neg. PW. The color scale shows the SHAP value of the relevant feature, shown on the *x*-axis. These highlights correlations found in the dataset as *J*_pk_ tends to increase at lower neg. PW, a result of power-control used during the sputter deposition.

To gain deeper insight into the underlying structure of the data, scatter plots of SHAP values are used to examine correlations, interaction effects, and general trends throughout this work. In Fig. 3a, a spread in the frequency is observed, depending on the neg. PW. In SHAP, this is called feature interaction; the features' importance depends on the value of another. More specifically, as the neg. PW increases, the impact of frequency decreases. This is seen as well in Fig. 3b, which gives an idea of the spread of data over the parameter space. Here, the neg. PW is plotted against frequency and its SHAP value on the color-scale.

In Fig. 3c a correlation is observed, with higher *J*_pk_ at lower neg. PW values. This comes from the use of power control during data sampling, which limits the voltage applied to the sputter target (and thus *J*_pk_). This was needed to keep *J*_pk_ below values where arcing or plasma instabilities are observed. The correlation of *J*_pk_ to neg. PW is mirrored in the frequency as well (shown in Fig. S8). Such correlations make it difficult to ascertain where interactions observed in Fig. 3a and b come from, as they can be a result of spurious attribution. In this case, the true interaction with frequency may be with *J*_pk_, but is mediated through the neg. PW because of underlying correlations in the dataset.

Correlations can sometimes be handled by conditional SHAP analysis techniques, such as permutation or partition explainers, which instead of applying the empirical mean to “missing” values when creating coalitions during SHAP analysis (*e.g.* exact explainer) will randomly permute missing features (permutation explainer) or sample expected values based on the created coalitions (partition explainer). We have found in the study of these datasets that conditional explanations, shown in Fig. S9, do not separate the interactions seen here between frequency and neg. PW. This may indicate some validity to those interactions as frequency and neg. PW together describe the off-time in the HiPIMS discharge, which is important for background plasma conditions relevant to the deposition rate.

### Visualization of back-attraction and plasma-ignition

Detailed SHAP analysis of parameters linked to high feature importance give insights into physics informed trends. As a reminder, and as described in the Experimental methods section, datasets were collected at discrete power densities to access a reasonable range of *J*_pk_ over a wide neg. PW range. The details of the boundary conditions and range accessible in each dataset are summarized in Table S1.

**Fig. 4:**
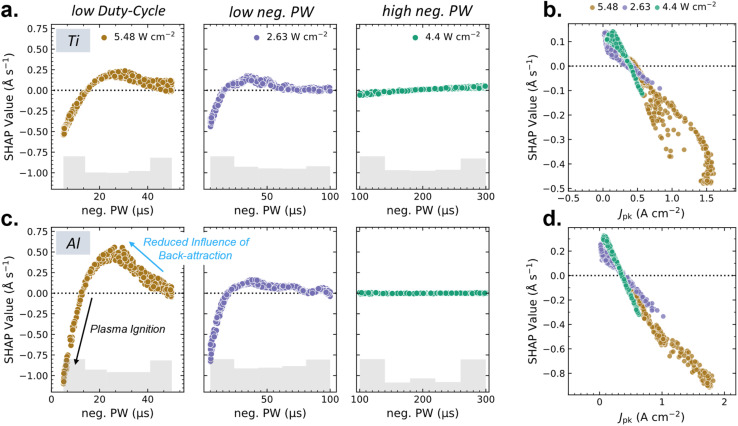
SHAP interpretations of the neg. PW (a and c) and *J*_pk_ (b and d) are shown for all six datasets collected in this work. This includes datasets for both Ti and Al collected at varying power densities (indicated by color) and neg. PW ranges; datasets are labeled on the top as low duty-cycle, low neg. PW, and high neg. PW for which additional information on their boundary conditions is included in Table S1. The model mean of each dataset is marked by a dotted line and the SHAP value on the *y*-axis indicates the predicted deviation from this mean. Three datasets of each (a) Ti and (c). Al are shown, where the SHAP interpretation of the neg. PW shows a notable increase in impact, attributed to the reduced influence of back-attraction. This is seen prominently in the low duty-cycle datasets. This prominence is attributed to the higher mean *J*_pk_ seen in the low duty-cycle dataset. The distribution of datapoints is visualized by the grey bar chart at the bottom of each graph. Additionally, a sharp decrease is observed at low neg. PW, attributed to energy loss during plasma ignition. As *J*_pk_ is an important feature for determining deposition-rates, we show for each (b) Al and (c) Ti the combined three datasets. SHAP-interpretation on this global dataset shows a near-linear trend with deposition rate.

In Fig. 4, we can see the SHAP scatter plots for all datasets involved in this study, covering >3000 process conditions. In Fig. 4a for Ti and Fig. 4c for Al, the influence of the neg. PW becomes prominent for datasets with a low neg. PW range (5–100 µs), with a visible peak around 25–30 µs for both Al and Ti datasets. This sudden increase in importance of the neg. PW is a known phenomenon in HiPIMS; it relates to the attraction of sputtered positive ions to the negatively charged sputter target, referred to as back-attraction. This causes a severe reduction in deposition-rates and is reported in several theoretical papers,^17,20,21,23^ as well as experimentally verified by Shimizu *et al.*^11^ When a voltage pulse ends, back-attraction ceases as the target potential returns to 0, and positively charged ions experience enhanced deposition-rates. This is seen as an increase in deposition-rate as pulses become shorter as the fraction of deposition happening after the voltage pulse ends becomes more significant. The increase in prominence of this effect for the low duty-cycle datasets is attributed to the higher *J*_pk_ and resulting higher ion counts, strongly affected by back-attraction. The mean *J*_pk_ of datasets is shown in Table S2 for reference. For the high neg. PW datasets, this is not observed as the reduced back-attraction pertaining to the end of the pulse becomes insignificant to the whole.

This peak is followed by a gradual, and subsequently sharp decrease at lower neg. PW. Indications of this relationship were seen experimentally by several groups^11,55^ and likely relate to the onset of plasma ignition, which can consume a significant amount of energy. This is interpreted by SHAP analysis as a negative correlation with the neg. PW, as energy spent on plasma ignition becomes proportionally more significant.

Additionally, spreading of the data noted in the low duty-cycle datasets was caused by the interaction of the neg. PW with the *J*_pk_ (see Fig. S10). As *J*_pk_ is an indicator of ion concentration, and the impact of back-attraction depends on this ion-concentration, it follows that these two features interact.

Visible as well in Fig. 4a and c for the low neg. PW datasets are minor oscillations within the 50–100 µs range. Although currently unexplained, the consistency of these features across many process points indicates a physical origin rather than statistical noise. As such, the complex plasma dynamics potentially underpinning this feature warrant further investigations.

Finally, for concise presentation of the data we choose to include a global overview of *J*_pk_ trends in Fig. 4b and d for Ti and Al datasets respectively, although individual SHAP analysis of *J*_pk_ in these datasets can be found in Fig. S11. This generalized overview of *J*_pk_ highlights the effectiveness of SHAP analysis at deconvoluting dominant process parameters such as power density or neg. PW. Deposition rates follow near-linear trends with *J*_pk_ for each individual power density.

### Local verification of trends indicated by SHAP

**Fig. 5:**
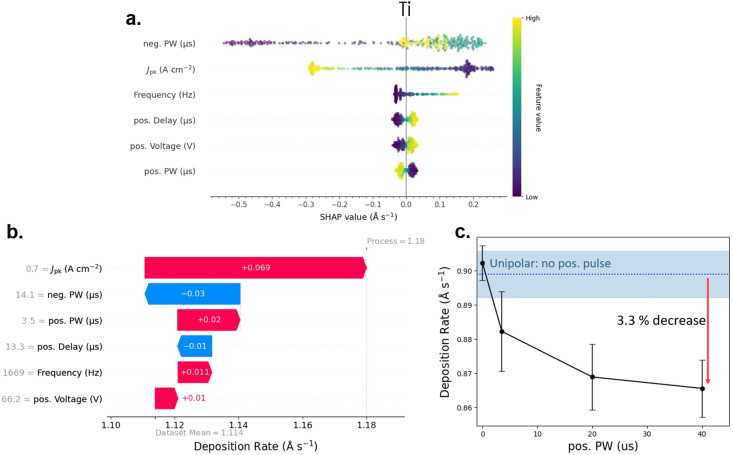
Above for Ti, and in Fig. S5 for Al, a dataset acquired under low duty-cycle (high *J*_pk_) conditions. Pos. pulse components become visibly more significant compared to the global analysis, as seen in the beeswarm plot (panel (a)). By comparing the SHAP values of the pos. pulse components with the absolute sum of SHAP values in individual processes, we identified processes where the pos. component is expected to have the highest impact. An example is shown in panel (b) as a waterfall plot of a process heavily impacted by pos. PW. Subsequently, measurements were manually performed while fixing all process parameters except the predicted most impactful pos. pulse parameter. An example for pos. PW is shown in panel (c) with the measurements for other pos. pulse parameters shown in Fig. S12. The dotted blue line indicates the measured deposition rate when all pos. pulse components were turned off, with the error bars and shaded blue area representing one standard deviation shaded. Overall, the trends in the pos. pulse components measured here align with those observed in the SHAP analysis, although deviate from the estimated linearity, likely due to the sparse sampling at the edge of the parameter space.

Finally, to target high *J*_pk_ regimes, a dataset was collected covering a range of ∼0.2–2 A cm^−2^. This was done by placing lower and upper bounds on the duty cycle of the process as opposed to the frequency, as described in the Experimental methods section. These targeted datasets for Al and Ti were collected, covering a range of neg. PW from 5–50 µs, with Ti shown in Fig. 5 and Al in Fig. S5, expecting the significance of pos. bipolar pulse components to increase in a higher *J*_pk_ dataset (and thus greater ionization of the process); and indeed, an increase of significance was observed, seen by the beeswarm plot in Fig. 5a or the side-by-side comparisons in Fig. S5. Note that changes in the importance of SHAP parameters are expected, as the exact explainers used in this work rely on empirical means when creating coalitions. As HiPIMS process parameters tend to interact heavily with *J*_pk_ (and thus ionization flux fraction), we have included in Table S2 the mean *J*_pk_ of each dataset, as a reference when visualizing these datasets.

In Fig. 5a, the beeswarm suggests a significant impact of the pos. pulse components. Namely, to improve deposition-rates over the model mean, the pos. delay and voltage should be increased, and the pos. PW decreased. This suggests that the pos. pulse components should be pushed far away from the HiPIMS pulse, and for the shortest duration possible, seen across all 6 datasets collected in this work (Fig. S5) and contradicts the conventional thinking that the pos. pulse should be placed immediately after the neg. pulse.

We theorize that the significance observed here (for the pos. delay and PW) relates to interactions with the background plasma conditions where significant deposition-rates have been observed for several hundred µs between HiPIMS pulses (*i.e.* during the afterglow), seen experimentally by Mitschker *et al.*^55^ Additionally, many groups report “quenching” of the plasma upon the application of a pos. pulse, noticed by onset delays in the rise of *J*_pk_^21^ as well as a drop in electron density of several orders of magnitude (over several 100 µs) after applying a positive pulse, effectively halting ionization and at times extinguishing the afterglow.^56^ These complex, and heavily process-dependent plasma dynamics may explain some of the contradictory reporting seen with bipolar HiPIMS deposition-rates. The dependence of background plasmas on bipolar pulses, magnetron balancing, and many other process parameters is a wide and complex field of study.^20,57–59^

Visualized in Fig. S12, we also note prominent interaction effects observed for each pos. pulse component (*e.g.* vertical spreading of the data) which arise from interactions with multiple different features and are quantified using a grid-based variance approach, summarized in Table S3.

By summing up the absolute SHAP values for an individual process, we can also rank which ones are most likely to be influenced by pos. pulse components. A local explanation of a process expected to be heavily impacted by pos. PW is shown as a waterfall plot in Fig. 5b. The different process parameters are displayed on the left, in order of importance, showing their expected contributions in increasing (red) or decreasing (blue) the measurements deviation from the model mean. In Fig. 5b, we can see that the pos. PW is estimated to increase the dep-rate by 0.02 Å s^−1^ from the model mean and is a significant component.

Several processes exhibiting a high predicted influence of pos. pulse components were selected for manual verification in this way. The local explanations of each of these processes are included in Fig. S13. Manual verification involved using the same parameters of the original process but sweeping the pos. pulse component of interest and measuring the deposition rates. Additionally, the process was run with all pos. pulse components turned off to get an idea of improvements over a pure unipolar case. For example, the local explanation shown in Fig. 5b shares the same process conditions as the manually measured deposition rates shown in Fig. 5c. In Fig. 5c, the pos. PW was swept from 0–40 µs noting a continual decrease in deposition rates, followed; previously attributed to quenching of the plasma afterglow. Each point was measured 3 times, with the error bars and the shaded blue area around the unipolar case representing one standard deviation. The decrease in deposition rate when compared to a unipolar process with the same process conditions (dotted blue line) is estimated to be 3.3%. Similar measurements can be found for pos. delay and voltage next to their local explanations in Fig. S13. Some improvements in deposition-rate are seen in Fig. S13 but were minimal and often within the error of the measurement.

In general, the trends observed in the beeswarm plot of Fig. 5a are conserved, with a need for high pos. delay, low pos. PW, and high pos. voltage to increase dep-rates. Overall, the analysis in Fig. 5 may indicate that improvements in deposition rate are possible, but minimal using a positive HiPIMS pulse. In most cases, the positive pulse appears to be detrimental to the deposition-rate when placed immediately after the neg. pulse. We hypothesize that this behavior arises from a combination of factors: a minor, and likely short-lived, ion-reflecting sheath near the target, which enhances the deposition rate of nearby ions; premature quenching of the afterglow which reduces the residual deposition; and lower background plasma densities as a result of plasma quenching, which increases energy losses during the ionization of process gases at the beginning of each pulse.

## Conclusion

By effectively and autonomously sampling a large parameter space of >3000 bipolar HiPIMS process conditions, we demonstrate a data-driven approach to understanding deposition-rate improvements in bipolar HiPIMS. Using SHAP analysis, we were able to produce powerful visualizations of physical HiPIMS mechanisms such as back-attraction and plasma ignition.

Minimal improvements in deposition rate were possible, but in most cases, the positive pulse appears to be detrimental when placed immediately after the neg. pulse. We hypothesize that this behavior arises from a combination of factors the most important likely being associated with a quenching of the afterglow which reduces the residual deposition. The lower background plasma densities also increase energy losses during the ionization of process gases at the beginning of each HiPIMS pulse. Further investigation is warranted to investigate these phenomena in detail.

Overall, the results show that autonomous sampling using *in situ* plasma and process diagnostics is a powerful tool to gain insight into modern plasma-based deposition processes, in highly complex parameter spaces. Comprehensive data sets facilitate process modelling in a field where results are often influenced by external factors which are hard to control and monitor, such as chamber geometry, magnetic field distribution or even precursor purity. The proposed workflow combining autonomous experimentation and interpretable ML is broadly applicable and can be expanded with relevant *in situ* materials characterization, paving the way for physically informed, data-driven advancements in coating technologies.

## Author contributions

A. W.: conceptualization, data curation, formal analysis, investigation, methodology, software, visualization, writing – original draft, writing – review & editing; N. R.: conceptualization, data curation, formal analysis, investigation, methodology, software, visualization, writing – original draft, writing – review & editing; J. S.: investigation, writing – review & editing; J. H.-S.: methodology, writing – review & editing; S. S.: conceptualization, funding acquisition, methodology, resources, supervision, writing – review & editing.

## Conflicts of interest

There are no conflicts to declare.

## Supplementary Material

DD-005-D6DD00063K-s001

## Data Availability

All raw data pertaining to this project including the code base used to interpret the data and create the figures presented in both the main text and supplementary information (SI) are freely available and uploaded to a Zenodo database. The code and anlysis is available at DOI: https://doi.org/10.5281/zenodo.18495504 and the raw data at DOI: doi.org/10.5281/zenodo.18495401. Supplementary information is available. See DOI: https://doi.org/10.1039/d6dd00063k.
