# Supplementary material for: Autonomous sampling and SHAP interpretation of deposition-rates in bipolar HiPIMS
Source: Digit Discov. 2026 Apr 27;5(5):2221–31. doi: 10.1039/d6dd00063k (PMC13112526; doi:10.1039/d6dd00063k)
Supplement: DD-005-D6DD00063K-s001 [file DD-005-D6DD00063K-s001.pdf]

Supplementary Information (SI) for Digital Discovery.
This journal is © The Royal Society of Chemistry 2026

Supporting Information: *Autonomous Sampling and SHAP Interpretation of Deposition-Rates in Bipolar HiPIMS*

Alexander Wieczorek[1,+], Nathan Rodkey[1,+,], Jan Sommerhäuser[1], Jason Hattrick-Simpers[2,3], Sebastian Siol[1,*]*

[1]Laboratory for Surface Science and Coating Technologies, Empa–Swiss Federal Laboratories for Materials Science and Technology, Ueberlandstrasse 129, Duebendorf CH-8600, Switzerland.

[2]Department of Materials Science and Engineering, University of Toronto, M5S 3E4, Toronto, ON, Canada.

[3]Acceleration Consortium, University of Toronto, M7A 2S4, Toronto, ON, Canada.

+ These authors contributed equally

** Corresponding authors: nathan.rodkey@empa.ch, sebastian.siol@empa.ch*

Keywords: HiPIMS, bipolar pulsing, SHAP, Autonomous, Bayesian Optimization

Table S1 – Each dataset was collected using power control, setting lower and upper bounds for some process conditions during the exploration phase. These are process conditions that were considered during the Bayesian optimization and whose uncertainty was minimized over those bounds. For *low neg. PW* and *high neg. PW* datasets, lower and upper bounds were set on the neg. PW, frequency, and pos. pulse components. For *low duty-cycle* datasets, lower and upper bounds were set on the neg. PW, duty-cycle, and pos. pulse components. In all cases J_{pk} was not considered during the exploration and its error not minimized. Instead it was added for post-analysis.

	**Boundary Conditions for Each Parameter (lower bound, upper bound)**							
**Dataset Label**	J_{pk} (A cm^{-2})	neg. PW (μs)	Frequency (Hz)	Duty Cycle (%)	pos. Voltage (V)	pos. Delay (μs)	pos. PW (μs)	Power Density (W cm^{-2})
Al – low duty-cycle	(0.28 – 1.82)	(5 – 50)	(240 – 7500)	(1.2 – 3.75)	(0 – 100)	(1.5 – 40)	(0 – 40)	5.48
Al – low neg. PW	(0.01 – 1.02)	(5 – 100)	(500 – 5000)	(0.25 – 50)	(0 – 100)	(1.5 – 40)	(0 – 40)	2.63
Al – high neg. PW	(0.05 – 0.63)	(100 – 300)	(200 – 800)	(2 – 24)	(0 – 100)	(1.5 – 40)	(0 – 40)	4.4
Ti – low duty-cycle	(0.25 – 1.60)	(5 – 50)	(240 – 7500)	(1.2 – 3.75)	(0 – 100)	(1.5 – 40)	(0 – 40)	5.48
Ti – low neg. PW	(0.01 – 0.82)	(5 – 100)	(500 – 5000)	(0.25 – 50)	(0 – 100)	(1.5 – 40)	(0 – 40)	2.63
Ti – high neg. PW	(0.05 – 0.57)	(100 – 300)	(200 – 800)	(2 – 24)	(0 – 100)	(1.5 – 40)	(0 – 40)	4.4

Table S2 – Empirical mean of peak-current density for Al and Ti low duty cycle datasets.

Datasets	Al – low neg. PW	Al – high neg. PW	Al – low duty-cycle	Ti – low neg. PW	Ti – high neg. PW	Ti – low duty-cycle
J_{pk} (A cm^{-2})	0.175	0.205	0.995	0.163	0.205	0.816

Table S3 – Grid-based variance is used to quantify interaction effects. Each feature is split into 6 bins and the weighted variance is evaluated across columns and rows. This is shown below for the SHAP explanation of a low duty cycle Ti dataset. The evaluated features (pos. pulse components) are shown on the left with the weighted variance across rows and columns shown with the corresponding features.

	**Variance Across this Feature**					
**Evaluated Feature**	J_{pk} (A cm^{-2})	neg. PW (µs)	Frequency (Hz)	pos. Voltage (V)	pos. Delay (µs)	pos. PW (µs)
pos. Voltage (V)	0.036	0.098	0.033	-	0.012	0.019
pos. Delay (µs)	0.016	0.032	0.028	0.037	-	0.009
pos. PW (µs)	0.023	0.030	0.030	0.012	0.017	-

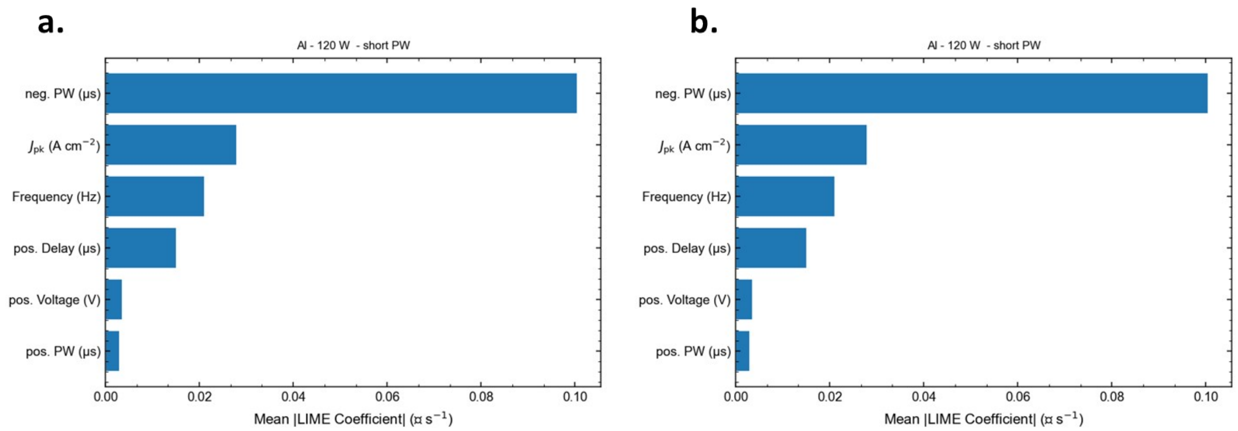

Figure S1. LIME analysis of all datasets is included in the GitHub repository, here an example of the Al, short PW dataset feature importance. Notably, it identifies the same dominant features described by the SHAP analysis later int this work.

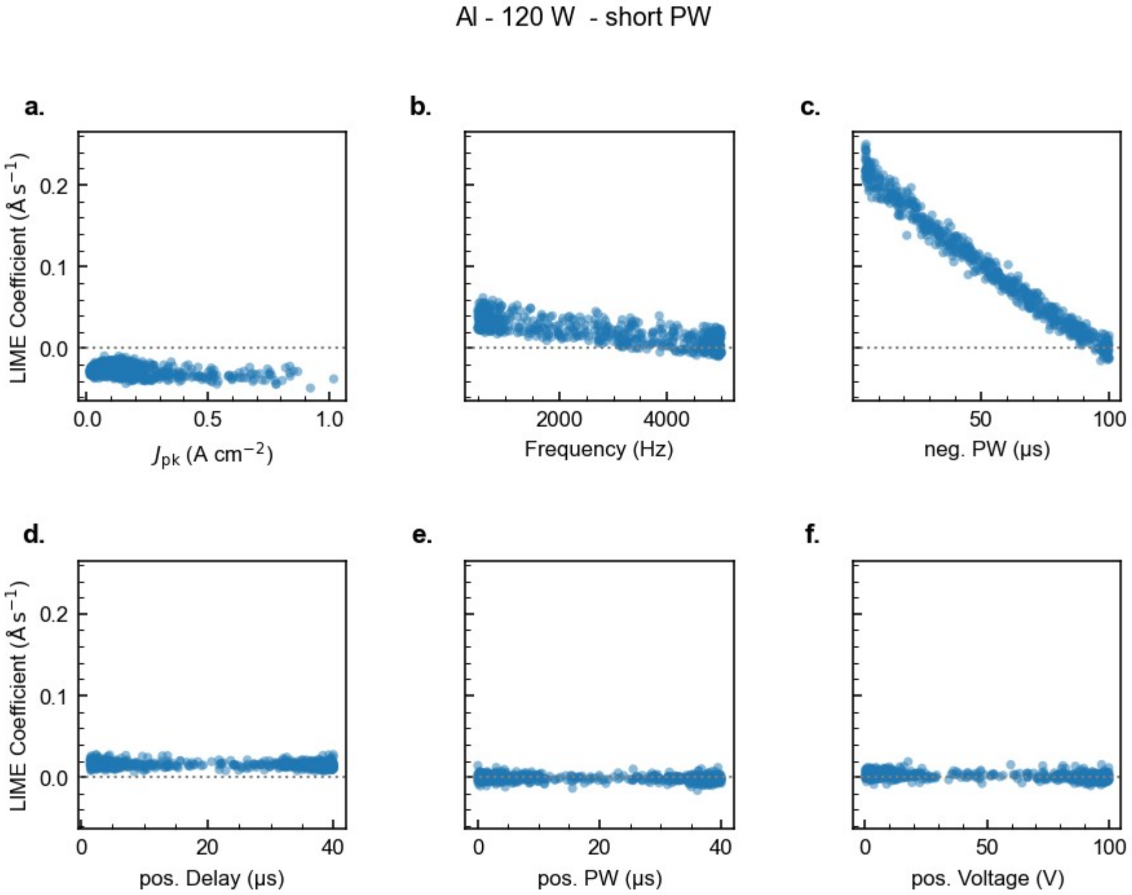

Figure S2. LIME analysis of all datasets is included in the GitHub repository, here an example of the Al, short PW dataset scatter plots, showing significant deviation from the SHAP analysis. Both sharp, and nonlinear features (in particular for the neg. PW relationship are no longer described by LIME. This is a common feature of LIME, which can reduce the interpretability of non-linear trends.

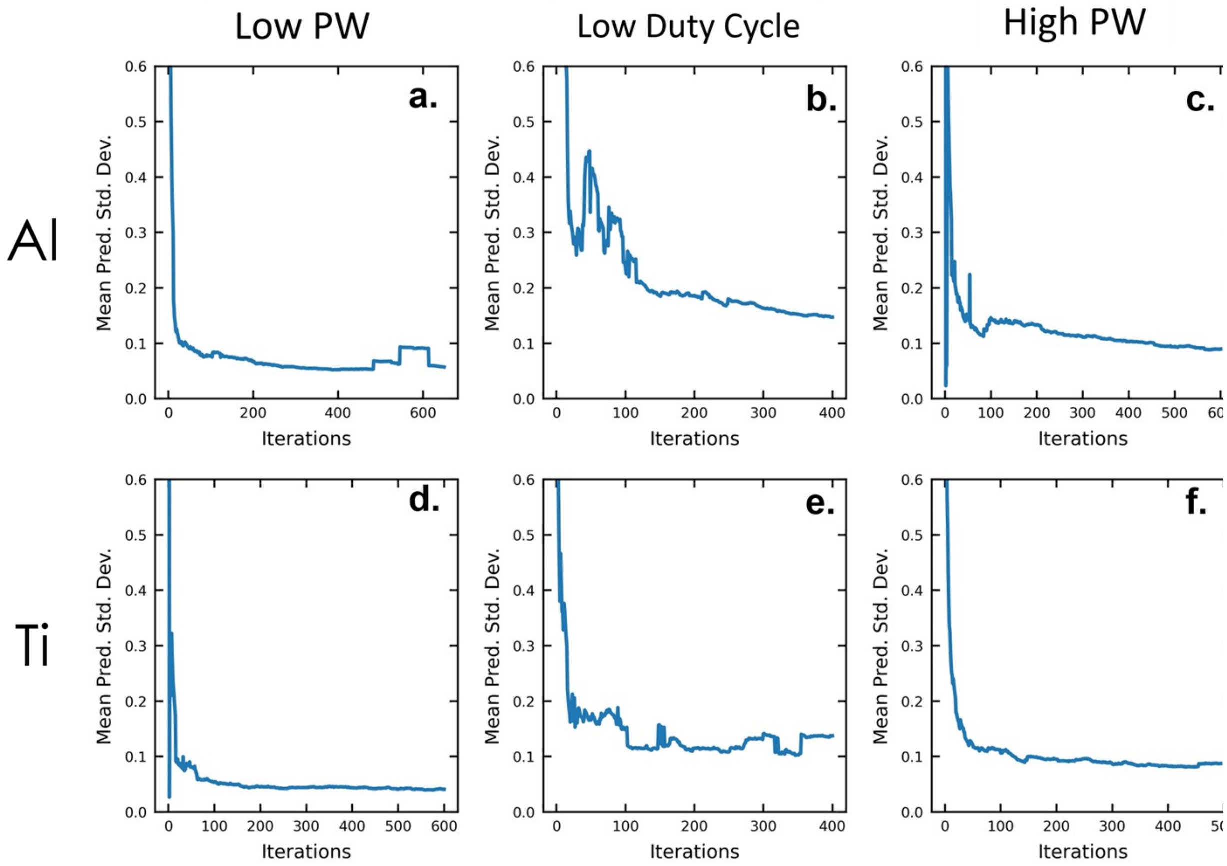

Figure S3. Mean standard deviations of datasets over iterations were evaluated by evenly splitting each feature into 5 bins to create testing data. Measurements of each dataset were then added manually, one at a time, evaluating the mean standard deviation across the testing data with the updated surrogate model. This is shown for Al (**a-c**) and Ti (**d-f**) datasets for the low pulse-width (**a,d**), low duty cycle (**b,e**), and high pulse-width (**c,f**) datasets, as described in the *Experimental Methods*. In general, the mean standard deviation is minimized after ~100 – 200 iterations, indicating high-quality sampling of the parameter space.

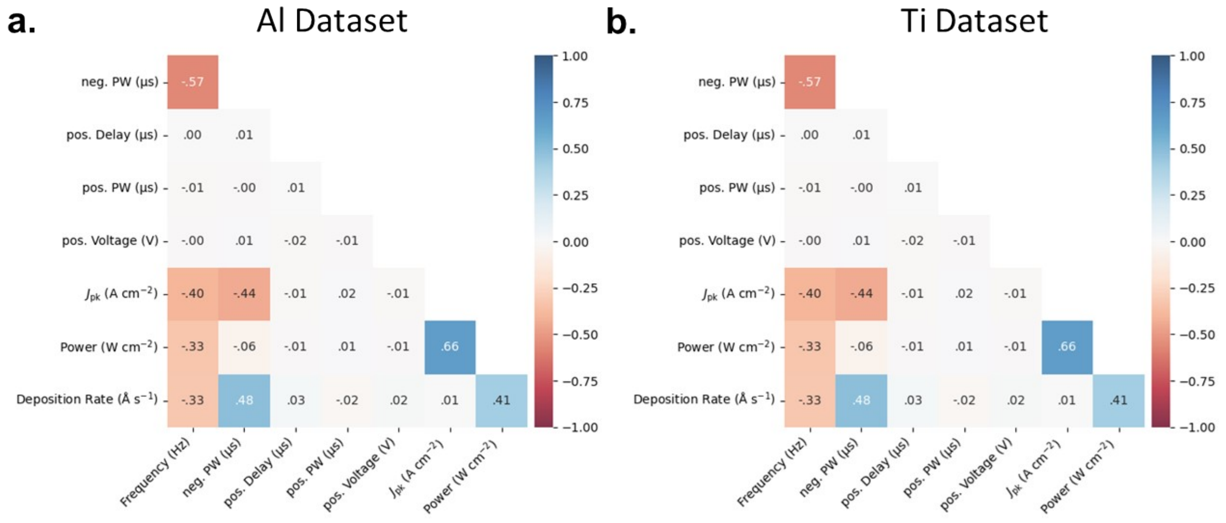

Figure S4. Spearman correlation matrices separated by metal-type for **a.** Al and **b.** Ti.

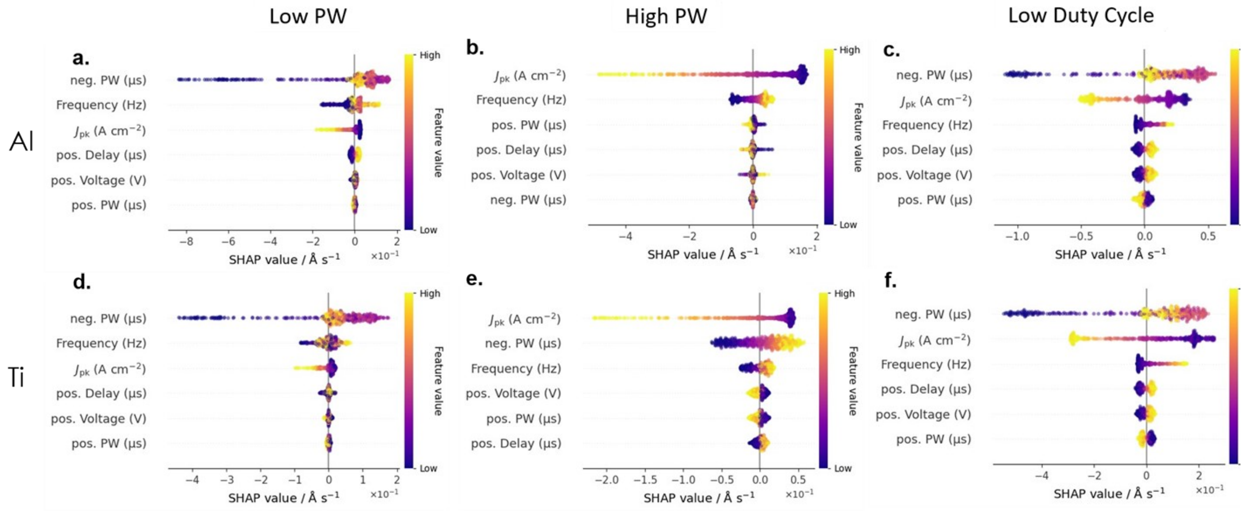

Figure S5. Beeswarm plots for all Al (**a-c**) and Ti (**d-f**) datasets are provided.

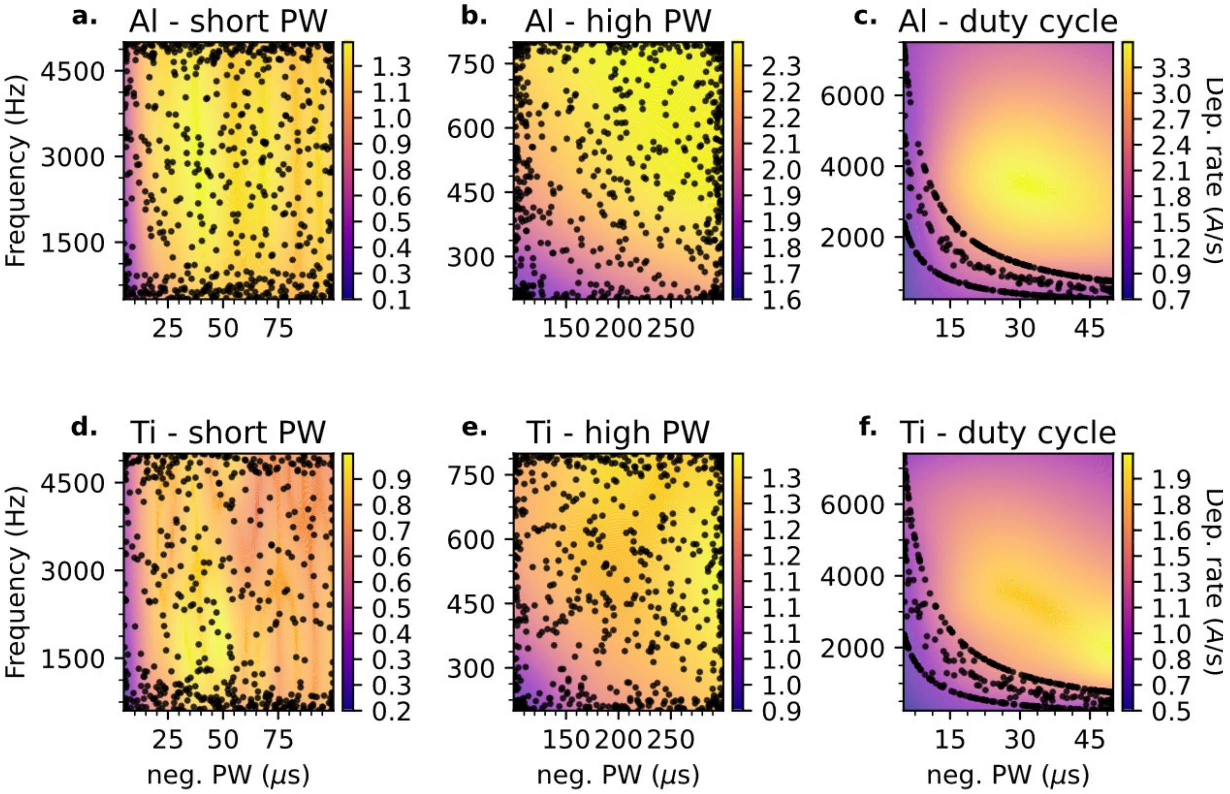

Figure S6. Surrogate model estimates of deposition rate as a function of negative pulse width and pulse repetition frequency for Al (**a–c**) and Ti (**d–f**) targets across three campaign configurations: short PW (120 W), high PW (200 W), and duty cycle series (250 W). The contour surfaces represent the Gaussian process posterior mean, with scattered points indicating measured parameter combinations.

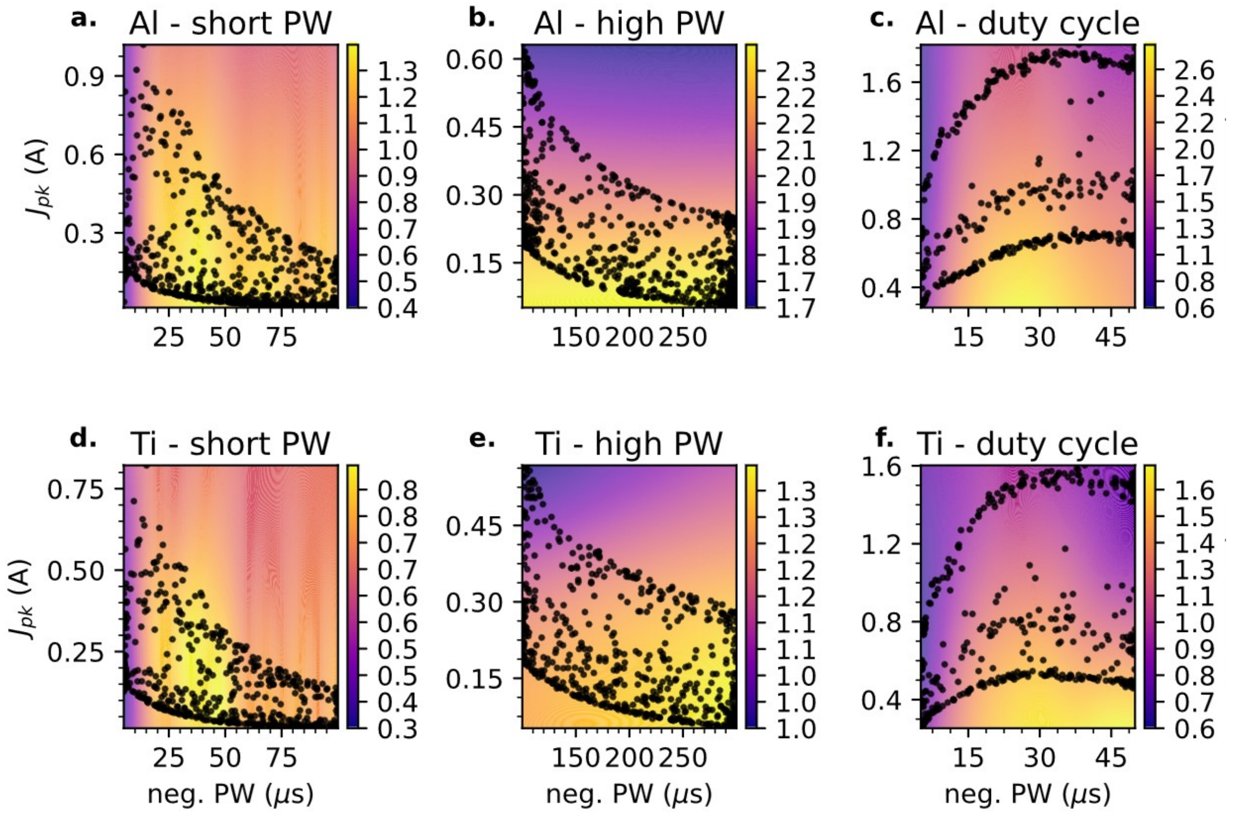

Figure S7. Surrogate model estimates of deposition rate as a function of negative pulse width and peak current density (J_{pk}) for Al (**a–c**) and Ti (**d–f**) targets across the same three campaign configurations. Including J_{pk} as an additional input dimension to the surrogate model reveals the relationship between achievable peak current and deposition rate across the pulse width parameter space.

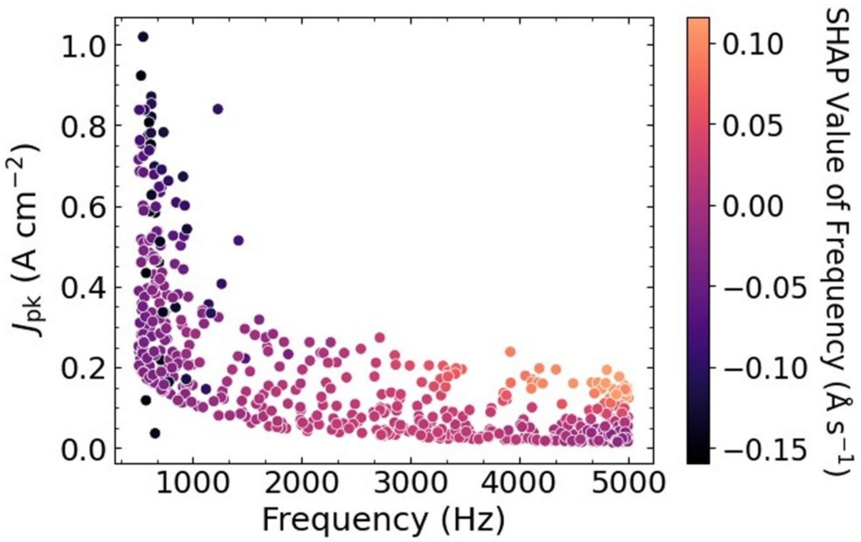

Figure S8. J_{pk} is plotted against Frequency, highlighting their correlation; higher J_{pk} values exist at lower frequency. This has to do with power control used during the sputter process, needed to maintain J_{pk} at values where arcing was not observed.

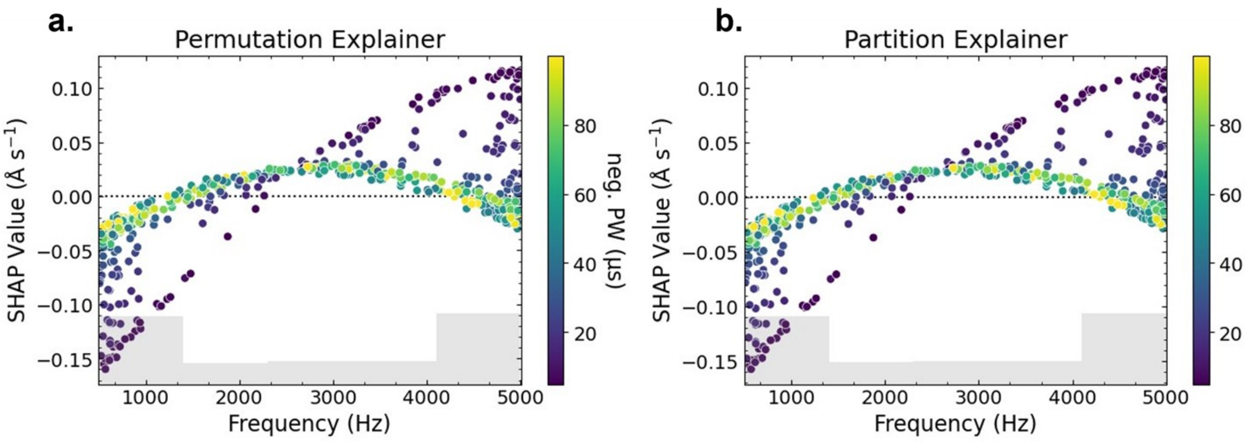

Figure S9. For the low pulse width, Al dataset presented in **Figure 3**, conditional SHAP explanations were evaluated to see if any interaction effects caused by correlated variables and spurious attributions could be resolved. In **a.** for a *Permutation Explainer* and **b.** a *Partition Explainer*. In both cases, the calculated SHAP values are nearly identical to the *Exact Explainer* used in the main body of this work, with only minute differences. This indicates the interaction effects observed here may have some validity, with *Frequency* representing the background plasma conditions of the process.

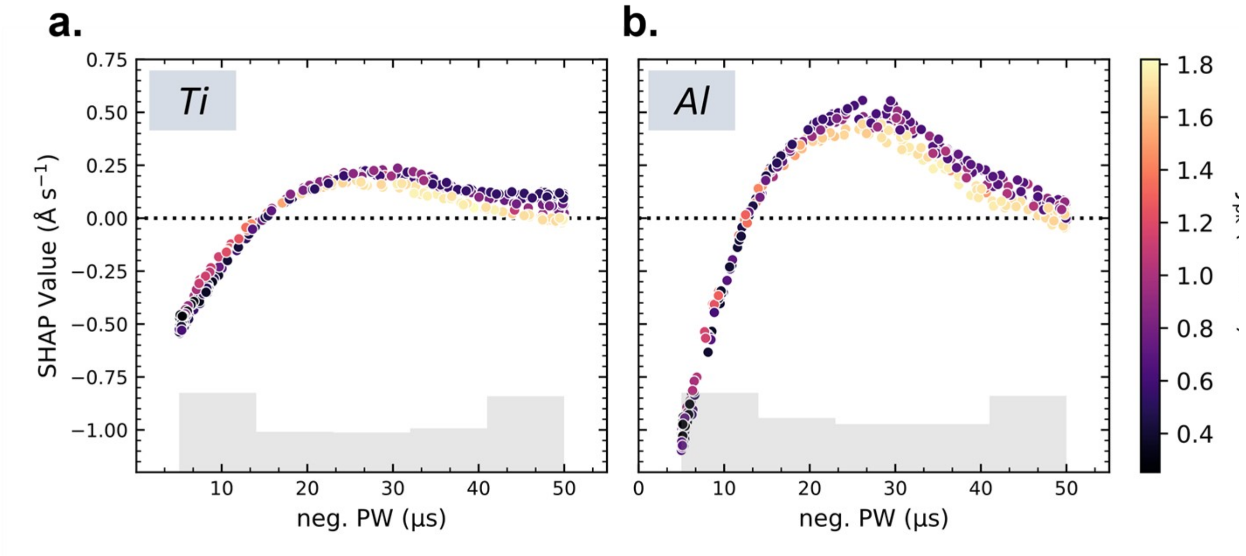

Figure S10. SHAP dependence plots for the low duty-cycle datasets of **a.** Ti and **b.** Al. The mode mean is indicated by a dotted line, and the distribution of data points is visible as a grey bar chart at the bottom of the graphs. A prominent peak is observed at 25 – 30 µs attributed to the reduced effect of back-attraction, as described in the main text. The color-scale indicates an interaction between the neg. PW and the J_{pk}. This interaction is expected as J_{pk} is an indicator for ion count, for which back-attraction plays a prominent role. Counter intuitively, as J_{pk} increases, the impact of the neg. PW decreases. This may be particular to the frame of the dataset, for show boundary conditions are described in greater detail in **Table S1**.

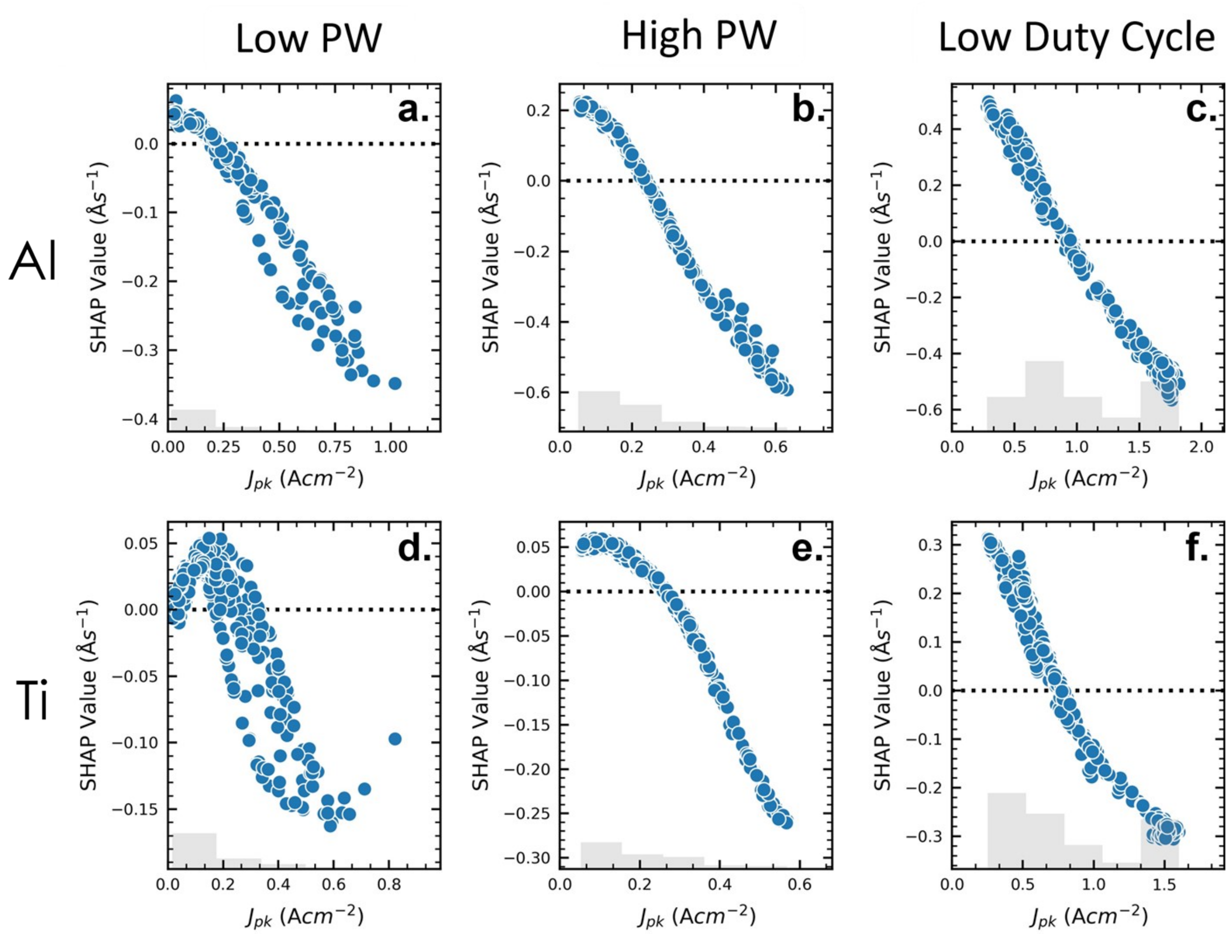

Figure S11. To better understand the expected relationships for individual datasets, and thus not include the potential distortion of effects caused by including heavily correlated features (e.g. Power Density), the SHAP scatter plots of J_{pk} are shown above for Al (**a-c**) and Ti (**d-e**) datasets. In most cases, a relatively linear relationship appears. Of interest, a noticeable s-shape is visible for a Ti dataset collected at neg. PW (5 – 100 μs) and is expected to be an artifact from insufficient data at the extremes of the J_{pk} range combined with overfitting.

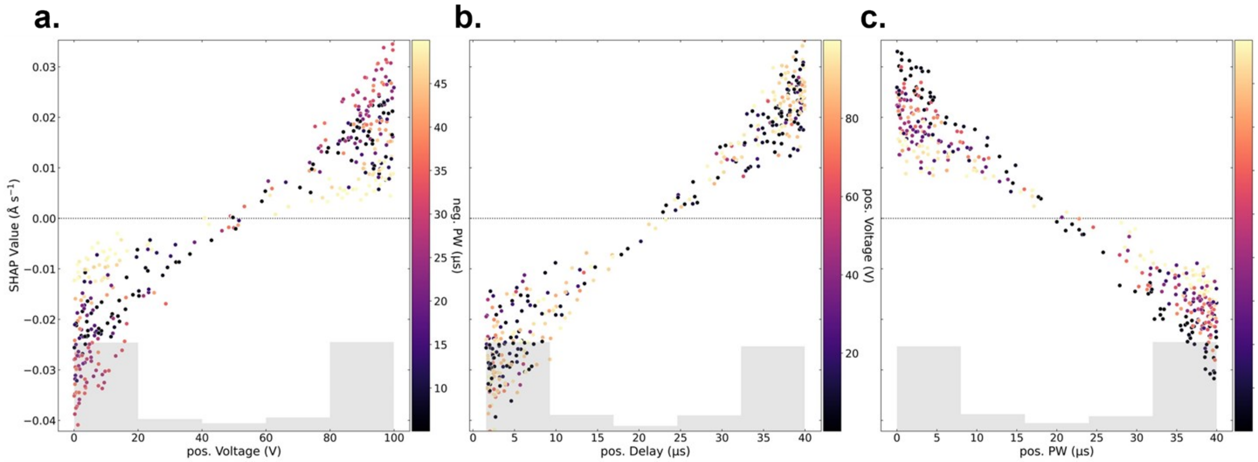

Figure S12. Scatter plots of the pos. pulse components for a Ti low duty-cycle dataset for **a.** pos. Voltage, **b.** pos. Delay, and **c.** pos. PW are shown above. The most prominent interaction effects (calculated with grid variance) of the pos. pulse components are plotted on the color-scale, with the quantified variance shown in **Table S1**. All parameters take on a bow-tie shape, common with parameters that are estimated to have low impact. The interactions are 'weak' and extrapolation of trends seen here cannot be done reliably. The distribution of datapoints is visualized by the grey bar chart at the bottom of each graph, with the mean of the dataset (0) indicated by a black dotted line.

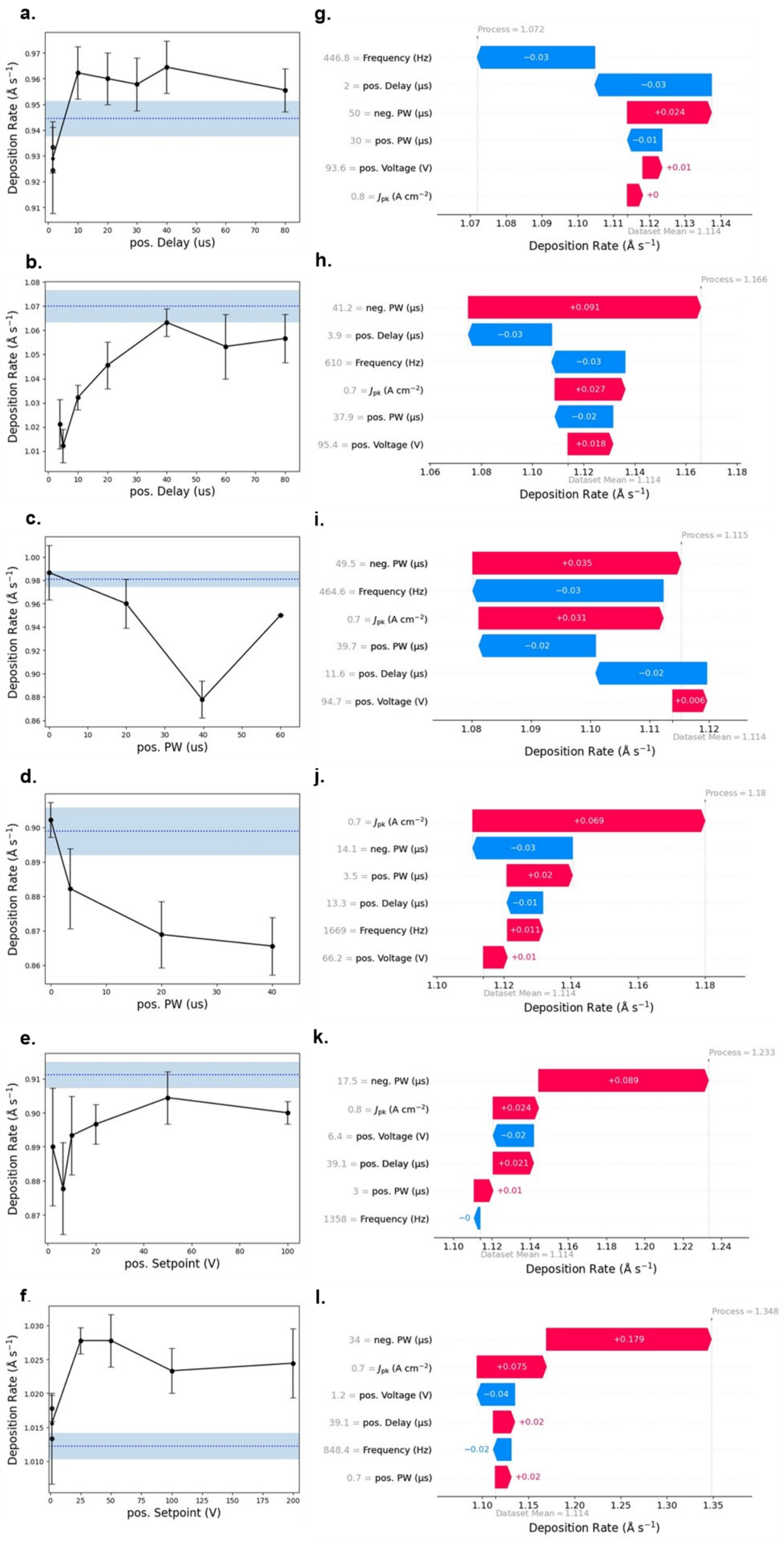

Figure S13. Individual process conditions were evaluated following the SHAP analysis. This was done by first evaluating where pos. pulse components were expected to have the most impact, done by comparing their SHAP values to the absolute sum of all SHAP values in the process. Two high-impact points were then chosen and their deposition rates measured while sweeping that parameter for pos. Delay (**a-b**), pos. PW (**c-d**) and pos. Voltage (**e-f**). The corresponding process conditions for each are shown to the right as waterfall plots in (**g-l**). The dotted blue line corresponds to the process condition where all pos. pulse components are turned off, to help evaluate increases in deposition rate to a purely unipolar case. In general, the trends follow those seen in the SHAP analysis, but lose their linearity, likely due to the sparse sampling conditions at the edge of the parameter space.
